# Supplementary material for: The prevalence of chronic kidney disease in people with severe mental illness: A systematic review protocol
Source: PLoS One. 2025 Jan 31;20(1):e0310568. doi: 10.1371/journal.pone.0310568 (PMC11785261; doi:10.1371/journal.pone.0310568)
Supplement: S1 Appendix — (DOCX) [file pone.0310568.s001.docx]

# Appendix 1 – MEDLINE Search Strategy

1. Exp Renal Dialysis/
2. h?emodialysis.tw,kf.
3. h?emofiltration.tw,kf.
4. h?emodiafiltration.tw,kf.
5. dialysis.tw,kf.
6. (Peritoneal adj1 (dialysis)).tw,kf.
7. (PD or CAPD or CCPD or APD).tw,kf.
8. Exp Renal Insufficiency/
9. Exp Kidney Failure/
10. Exp Renal Insufficiency, Chronic/
11. Exp Kidney Diseases/
12. Uremia/
13. (endstage adj1 (renal or kidney)).tw,kf
14. (ESRF or ESKF or ESRD or ESKD).tw,kf
15. (chronic adj1 (kidney or renal)).tw,kf
16. (CKF or CKD or CRF or CRD).tw,kf.
17. (predialysis).tw,kf.
18. (renal adj1 (transplant* or graft)).tw,kf.
19. (kidney adj1 (transplant* or graft)).tw,kf.
20. (1 OR 2 OR 3 OR 4 OR 5 OR 6 OR 7 OR 8 OR 9 OR 10 OR 11 OR 12 OR 13 OR 14 OR 15 OR 16 OR 17 OR 18 OR 19)
21. Exp Schizophrenia/
22. Exp Affective disorders, psychotic/
23. Exp Bipolar disorder/
24. (bipolar adj1 (disorder* or disease* or illness*)).tw,kf.
25. paranoid disorders/
26. Exp psychotic disorders/
27. schizo*.tw,kf.
28. (mani* adj3 depress*).tw,kf.
29. (psychotic* adj3 depress*).tw,kf.
30. (severe* adj3 affective*).tw,kf.
31. (severe* adj3 mental*).tw,kf.
32. (severe* adj3 depress*).tw,kf.
33. (psychos#s adj3 depress*).tw,kf.
34. (serious* adj3 affective*).tw,kf.
35. "serious mood*".tw,kf.
36. (serious* adj3 mental*).tw,kf.
37. (serious* adj3 depress*).tw,kf.
38. “Severe mental*”.tw,kf.
39. Severe adj1 (mental* or depress*).tw,kf.
40. “Schizoaffective disorder”.tw,kf
41. Exp Antipsychotic Agents/
42. Exp Antimanic Agents/
43. Exp Psychotropic Drugs/
44. (21 OR 22 OR 23 OR 24 OR 25 OR 26 OR 27 OR 28 OR 29 OR 30 OR 31 OR 32 OR 33 OR 34 OR 35 OR 36 OR 37 OR 38 OR 39 OR 40 OR 41 OR 42 OR 43)
45. Exp epidemiologic studies/
46. Exp epidemiology/
47. epidemiolog*.tw,kf.
48. Exp prevalence/
49. prevalence.tw,kf.
50. Exp incidence/
51. incidence.tw,kf.
52. Exp Observational Study/
53. observational.tw,kf.
54. Longitudinal Studies/
55. longitudinal.tw,kf.
56. Case-Control Studies/
57. Exp Cross-Sectional Studies/
58. Exp Cohort Studies/
59. “cohort”.tw,kf.
60. Exp Risk/
61. Exp Risk Factors/
62. (45 OR 46 OR 47 OR 48 OR 49 OR 50 OR 51 OR 52 OR 53 OR 54 OR 55 OR 56 OR 57 OR 58 OR 59 OR 60 OR 61)
63. (20 AND 44 AND 62)
